# The biological behavior and clinical outcome of pituitary adenoma are affected by the microenvironment

**DOI:** 10.1111/cns.14729

**Published:** 2024-05-13

**Authors:** Yuhe Li, Xiufang Ren, Wei Gao, Ruikai Cai, Jianqi Wu, Tianqi Liu, Xin Chen, Daoming Jiang, Chong Chen, Quan Cheng, Anhua Wu, Wen Cheng

**Affiliations:** ^1^ Department of Neurosurgery Shengjing Hospital of China Medical University Shenyang Liaoning China; ^2^ Department of Pathology Shengjing Hospital of China Medical University Shenyang Liaoning China; ^3^ Shenyang ShenDa Endoscopy Co., Ltd. Shenyang Liaoning China; ^4^ Department of Neurosurgery, Xiangya Hospital Central South University Changsha Hunan China; ^5^ National Clinical Research Center for Geriatric Disorders, Xiangya Hospital Central South University Changsha Hunan China

**Keywords:** biological behavior, outcome, pituitary adenoma, refractory pituitary adenoma, tumor microenvironment

## Abstract

**Background:**

Pituitary adenoma is one of the most common brain tumors. Most pituitary adenomas are benign and can be cured by surgery and/or medication. However, some pituitary adenomas show aggressive growth with a fast growth rate and are resistant to conventional treatments such as surgery, drug therapy, and radiation therapy. These tumors, referred to as refractory pituitary adenomas, often relapse or regrow in the early postoperative period. The tumor microenvironment (TME) has recently been identified as an important factor affecting the biological manifestations of tumors and acts as the main battlefield between the tumor and the host immune system.

**Main body:**

In this review, we focus on describing TME in pituitary adenomas and refractory pituitary adenomas. Research on the immune microenvironment of pituitary adenomas is currently focused on immune cells such as macrophages and lymphocytes, and extensive research and experimental verifications are still required regarding other components of the TME. In particular, studies are needed to determine the role of the TME in the specific biological behaviors of refractory pituitary adenomas, such as high invasion, fast recurrence rate, and high tolerance to traditional treatments and to identify the mechanisms involved.

**Conclusion:**

Overall, we summarize the similarities and differences between the TME of pituitary adenomas and refractory pituitary adenomas as well as the changes in the biological behavior of pituitary adenomas that may be caused by the microenvironment. These changes greatly affect the outcome of patients.

## Graphical abstract

The components of the pituitary adenoma microenvironment are complex and interrelated, including types of immune cells, extracellular matrix, microvasculature, etc. Here, we summarize the similarities and differences between the microenvironment of pituitary adenomas and refractory pituitary adenomas, highlighting the important role of microenvironment in determining biological behavior and clinical outcomes.

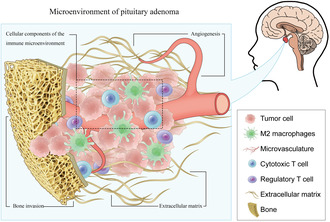

## 1 INTRODUCTION

Pituitary adenoma is one of the most common brain tumors, accounting for 10%–25% of all brain tumors, and most patients can be treated with surgery and/or drugs.^1^ However, refractory pituitary adenoma has a fast growth rate and is resistant to conventional treatment, such as surgery, drug therapy, and radiation therapy, and often relapses or regrows in the early postoperative period.^2^, ^3^, ^4^, ^5^, ^6^ The clinical consequences of refractory pituitary adenoma are serious, such as tumor growth, cranial nerve compression, bone destruction and invasion, and excessive hormone secretion, which seriously affect the health of patients.

One of the main factors affecting the biological behavior of tumors is the tumor microenvironment (TME), which also plays an important role in the fight between the tumor and the host.^7^, ^8^, ^9^, ^10^ Current research on the TME has mainly focused on the immune cells, especially tumor‐related macrophages^11^, ^12^; however, nonimmune cells and noncellular components also play important roles in the immune microenvironment.^13^ There are many similarities and differences in the immune microenvironment between refractory pituitary adenoma and common pituitary adenoma. These factors affect the occurrence, development, and prognosis of pituitary adenoma and refractory pituitary adenoma and determine the treatment strategy of different types of pituitary adenoma to a certain extent.

Refractory pituitary adenoma and common pituitary adenoma have many similarities and differences in the tumor immune microenvironment, which contribute to the special biological characteristics of refractory pituitary adenoma, such as strong invasion ability, early postoperative recurrence, stronger drug resistance, and so on. This review therefore considers the immune microenvironment and then explores the reasons for the invasiveness, drug resistance, and postoperative recurrence of pituitary adenomas, especially refractory pituitary adenomas, and the factors affecting the immune microenvironment of pituitary adenomas.

## 2 DEFINITION OF REFRACTORY PITUITARY ADENOMA

According to the 2017 WHO Classification of pituitary adenomas and the Expert Consensus on Diagnosis and Treatment of refractory pituitary adenomas in China (2019), the definition of refractory pituitary adenomas is summarized as follows: Refractory pituitary adenoma shows aggressive growth on imaging, and its growth rate is faster than that of general pituitary adenoma. It is resistant to conventional treatment, such as surgery, drug therapy, and radiation therapy, and often relapses or regrows in the early postoperative period.^3^, ^5^ The 2017 WHO Classification of pituitary adenomas emphasizes that refractory pituitary adenomas have the following five characteristics: I. Invasive growth. II. Rapid growth. III. After multimodality standardized treatment such as surgery, drugs, and radiation therapy, tumors continue to grow and/or secrete excessive hormones. IV. Tumor enlargement and/or high levels of hormone secretion seriously affect patients' quality of life and threaten their lives. V. The prognosis of the patient is poor, but intracranial and distant metastasis of the tumor are not present^5^ (Table 1).

**TABLE 1:** Summary of characteristics of refractory pituitary adenoma.

Characteristics of refractory pituitary adenoma	
I	Invasive growth
II	Rapid growth
III	After multimodality standardized treatment such as surgery, drugs, and radiation therapy, tumors continue to grow and/or secrete excessive hormones
IV	Tumor enlargement and/or high levels of hormone secretion seriously affect patients' quality of life and threaten their lives
V	The prognosis of the patient was poor, but intracranial and distant metastasis of the tumor was not present

Most refractory pituitary adenomas are aggressive. Invasiveness is also an important biological feature and a key factor in the poor prognosis of refractory pituitary adenomas. In addition, invasive pituitary adenoma has a higher risk of tumor progression, which is one of the main reasons why surgical resection is difficult. It is worth mentioning that not all invasive pituitary adenomas are refractory pituitary adenomas, but tumor invasiveness is indeed one of the main clinical features of refractory pituitary adenomas. Therefore, in reviewing the effects of TME on the biological behavior of refractory pituitary adenomas and the differences between refractory and common pituitary adenomas, we will focus on the factors related to tumor aggressiveness.

## 3 CHANGES IN THE TUMOR IMMUNE MICROENVIRONMENT MAY CAUSE PITUITARY ADENOMAS TO DEVELOP A MORE “MALIGNANT” PHENOTYPE

Refractory pituitary adenoma is defined as a tumor with aggressive growth on imaging. However, the definition of refractory pituitary adenoma is not clear, and there is no specific pathological classification at present.^14^, ^15^ This lack of clarity means that invasive pituitary adenomas, atypical pituitary adenomas, or pituitary carcinomas may have been included in previous studies.^3^, ^4^, ^5^, ^16^, ^17^ Refractory pituitary adenomas are reported to occur in about 10%–45% of clinically detected pituitary adenomas. According to statistics, about 0.5% of pituitary adenomas have an invasive course. Invasive behavior is an important biological behavior of refractory pituitary adenoma.^2^, ^18^, ^19^, ^20^, ^21^, ^22^, ^23^ The microenvironment is often highly correlated with the aggressiveness of the tumor.^24^, ^25^, ^26^ It is therefore important to understand the immune microenvironment of refractory pituitary adenomas.^27^

## 4 CELLULAR COMPONENTS OF THE IMMUNE MICROENVIRONMENT

### 4.1 Macrophage

**FIGURE 1:**
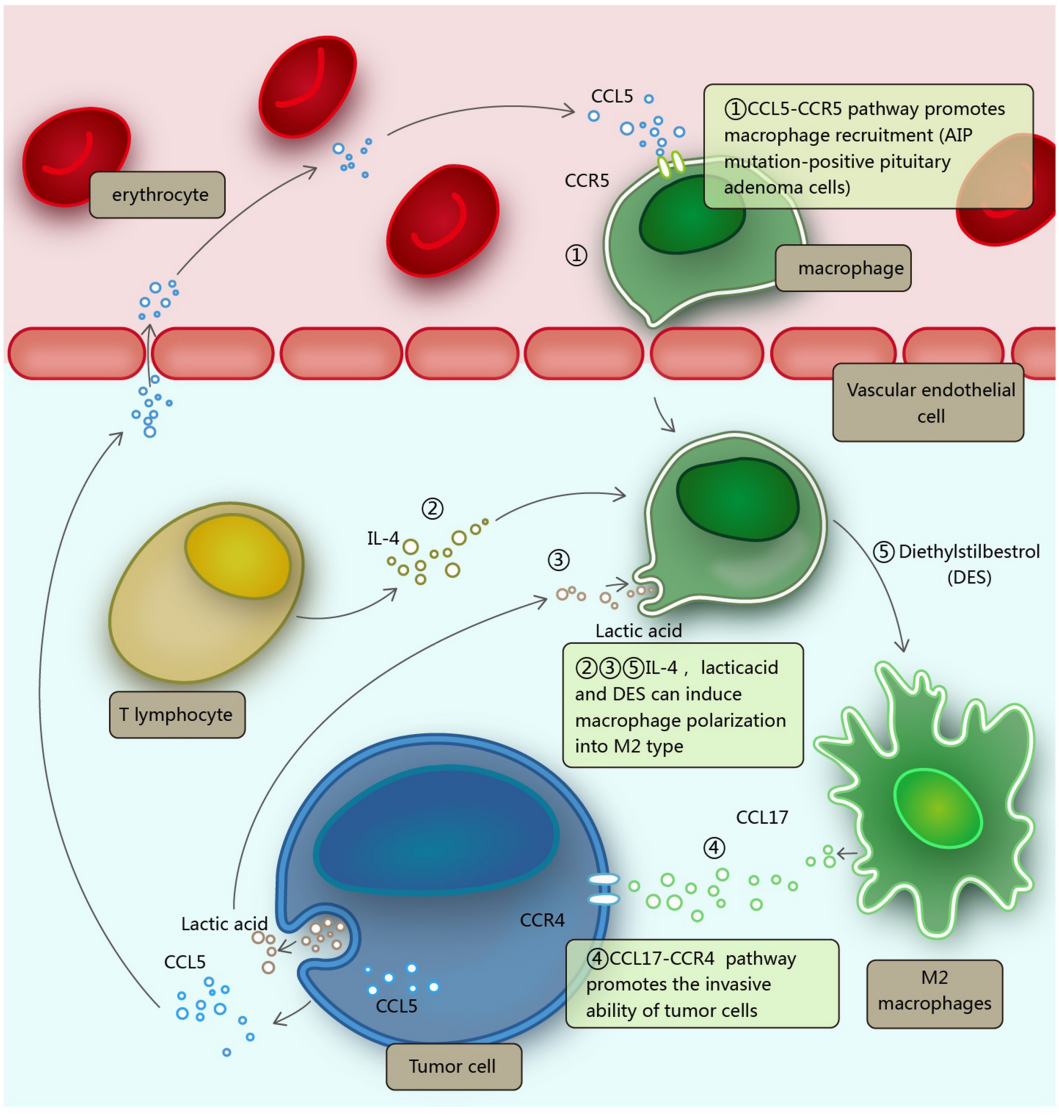
Macrophages in the immune microenvironment of pituitary adenoma: Procedure ①: Macrophage recruitment is promoted through the CCL5–CCR5 pathway, which mainly occurs in AIP mutation‐positive pituitary adenomas^31^; Procedures ②, ③, ⑤: IL‐4, lactic acid, and DES can induce macrophages to polarize into M2 phenotype^30^, ^33^, ^43^; Procedure ④: M2‐type macrophages can improve the invasion ability of tumor cells through the CCL17–CCR4 pathway.^63^

Macrophages are usually the most abundant group of immune cells in the TME,^11^, ^28^, ^29^ including in pituitary adenomas. Patients with pituitary adenomas generally show high levels of macrophage infiltration in the pituitary glands.^30^, ^31^, ^32^ Two studies have successively confirmed the presence of M1 and M2 macrophages in estrogen‐induced prolactinoma pituitary (Figure 1. Procedure ⑤) and nonfunctioning pituitary adenomas.^33^, ^34^ In addition, single‐cell transcriptome and single‐cell multiomics analyses revealed that macrophages and T lymphocytes were abundant in pituitary adenomas (most of the cells were still tumor cells). Immunohistochemical staining also confirmed the validity of the single‐cell sequencing results.^35^ In addition, the ratio of the M2 marker CD163 to the M1 marker HLA‐DR was about three times higher in pituitary adenomas than in normal pituitary tissue, indicating a higher proportion of M2‐type macrophages in pituitary adenomas.^30^ In addition, levels of the M2 polarization factor interleukin (IL)‐4 have been reported to be about five times higher in pituitary adenomas compared with levels of the M1‐type factor interferon (IFN)‐γ, and the ratio corresponds to the M2/M1 cell ratio^30^ (Figure 1, Procedure ②). Other studies reached similar conclusions.^36^, ^37^ Tumor‐associated macrophages have been shown to play many different roles in the TME to promote tumor progression.^38^, ^39^, ^40^, ^41^ In turn, pituitary adenomas may also have a special pathway to stimulate the migration of macrophages to the region where the tumor cells are located, which further affects the composition of the immune microenvironment within the tumor^31^ (Figure 1, Procedure ①). According to the above results, it can be inferred that M2 macrophages may also play a role in the occurrence and development of pituitary adenomas.^33^

Significantly enhanced invasion ability is one of the important characteristics of refractory pituitary adenoma, and macrophages can affect the invasion ability of pituitary adenoma in many ways. *AIP* mutation‐positive pituitary adenomas include large numbers of infiltrating macrophages, and further studies showed that the role of normal *AIP* expression in maintaining the normal state of pituitary adenomas appears to be important, as positive or low *AIP* mutation expression is indicative of a more aggressive pituitary adenoma phenotype^31^, ^42^ (Table 2). Further studies have explored the specific mechanism by which macrophages affect the invasion ability of pituitary adenoma: on one hand, pituitary adenomas cause acidification of the TME, resulting in polarization of tumor‐related macrophages into an M2‐like phenotype (Figure 1, Procedure ③), whereas on the other hand, M2‐like macrophages secrete CCL17 and enhance the invasion ability of pituitary adenoma cells via the CCL17/CCR4/mammalian target of rapamycin complex 1 axis^43^ (Figure 1, Procedure ④). Other studies have shown similar results. In a subtype of nonfunctional pituitary adenoma, macrophages were recruited and polarized into an M2‐like phenotype, a process that increased the invasion ability of this subtype of nonfunctional pituitary adenoma and the proliferation of tumor cells.^34^ However, functional pituitary adenomas and gonadotropin tumors can also recruit macrophages and subsequently convert them into M2‐like phenotypes, and an analysis of clinical data confirmed that the percentage of M2‐like macrophages was significantly correlated with the aggressiveness of gonadotropin pituitary adenomas.^37^ Not only that, other studies have suggested a positive correlation between the degree of infiltration of macrophages and the size of pituitary adenomas, with larger adenomas containing more macrophages.^32^

**TABLE 2:** Distinction between refractory pituitary adenoma and pituitary adenoma (1).

	Refractory pituitary adenoma	Pituitary adenoma
Cellular level		
Immune cell		
Macrophage	Obvious presence and infiltration	Mainly M2 macrophages
T lymphocyte	Tregs were associated with invasiveness	Obvious presence
Natural killer cell	CD56+ cell (NK cell) levels of invasive NFPA were reduced	NK‐1‐positive cells were common
Tumor‐associated fibroblasts	Significantly promoted the growth of pituitary cells in rats	–
Molecular level		
Macrophage related		
AIP	AIP mutation positive or AIP low expression	Normal expression
CCL17	Upregulation	NA
T lymphocyte related		
IL‐10	High expression	expression
IL‐4/IL‐5/IL‐17	Postoperative decrease	
Th1/Th2 ratio	Postoperative decrease	
IFN‐γ/TNF‐α	Postoperative elevation	
ARG‐1	Upregulated and inhibited T lymphocyte function	NA
COX‐2	Up‐regulated and inhibited T lymphocyte proliferation	NA
Tumor cell‐related		
PD‐1/PD‐L1	High expression	The expression level was lower than that of invasive pituitary adenoma

In summary, macrophages may affect the function of pituitary adenoma cells in various ways, and ultimately lead to the enhancement of invasion and proliferation ability of refractory pituitary adenoma. However, there are still no studies to investigate the relationship between refractory pituitary adenoma and macrophages in the immune microenvironment. However, according to the above, we hypothesized that the polarization and enrichment of macrophages may be closely related to the formation of refractory pituitary adenoma.

### 4.2 T lymphocyte

T cells play a key role in tumor immunity, and the degree of their invasion in tumors is closely related to tumor prognosis.^44^, ^45^ Recent studies highlighted that regulatory T lymphocytes and cytotoxic T lymphocytes are the major T cell types with play a very important and almost opposite role in tumor immunity. Therefore, in the following review, T lymphocytes in the pituitary adenoma microenvironment were discussed around these two cell groups.

#### 4.2.1 Regulatory T lymphocytes

By suppressing antitumor immunity, regulatory T cells (Tregs) can prevent protective immunity from monitoring tumors. Thus, the body loses the effective antitumor immune response. Eventually, this process will promote the development of tumors.^46^, ^47^ In most cancers, a high ratio of Tregs/CD8 + T cells usually indicates a poor prognosis.^48^ In pituitary adenomas, studies have found that more aggressive and poor prognostic adenomas tend to have significant upregulation of Treg cells.^49^, ^50^

#### 4.2.2 Cytotoxic T lymphocytes

In general, cytotoxic T cells target host cells infected by viruses or cancerous cells. They induce apoptosis of target cells by secreting perforin and granzyme. However, the effect of cytotoxic T lymphocytes on pituitary adenoma is still controversial. For example, silent type III pituitary adenomas with less cytotoxic T lymphocyte infiltration have a higher risk of recurrence and invasion.^51^ Another study of growth hormone‐secreting pituitary adenomas showed that the accumulation of cytotoxic T lymphocytes (CD8 + T cells) in the pituitary adenoma microenvironment was negatively correlated with invasive behavior and drug resistance. This result is consistent with the effect of cytotoxic T cells on pan carcinoma.^52^

**FIGURE 2:**
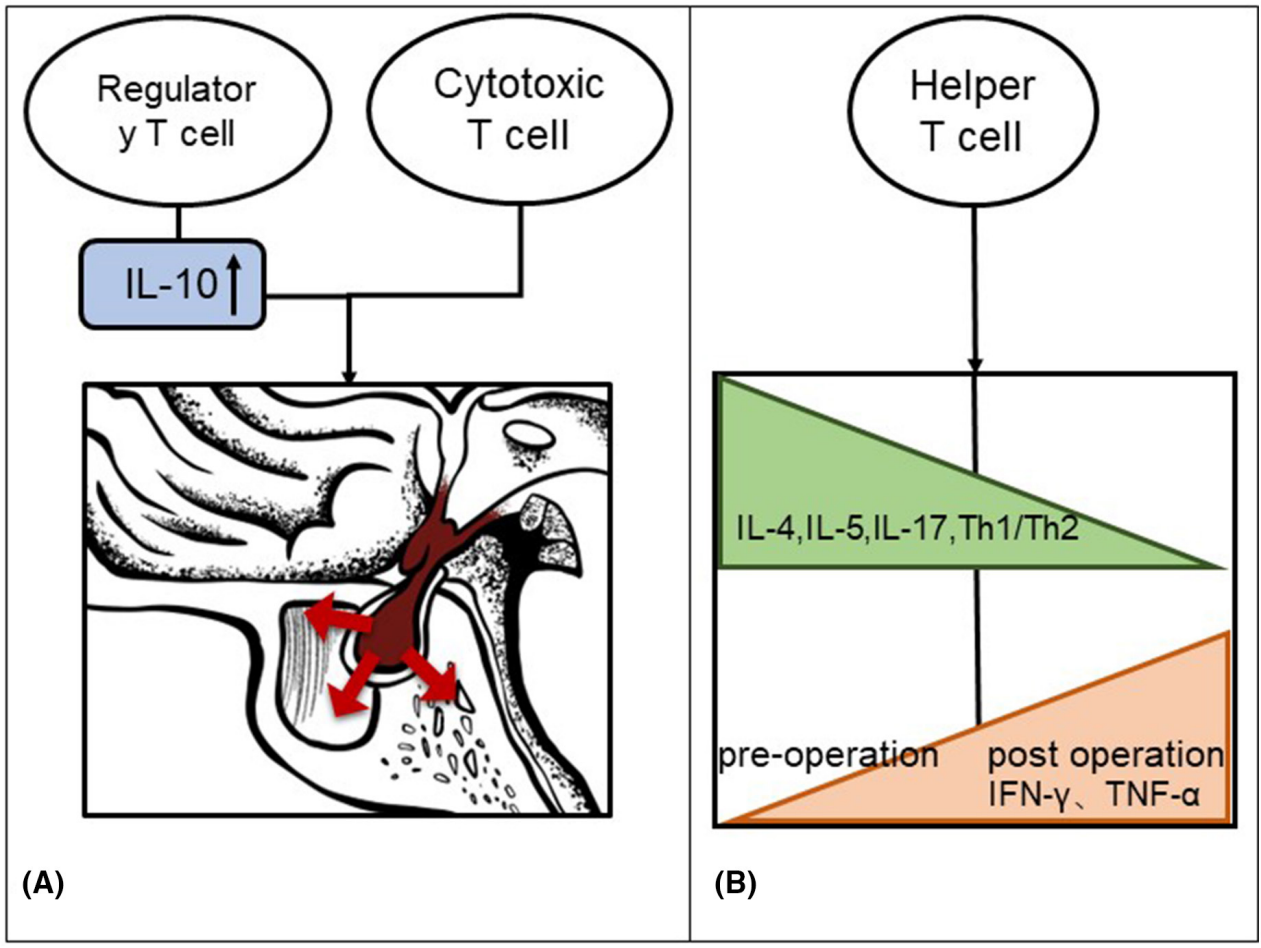
Effect of T lymphocytes on immune microenvironment of pituitary adenoma. (A) Regulatory T cells and cytotoxic T cells may enhance the invasion ability of pituitary adenoma; (B) Preoperative and postoperative changes of TH cell‐associated cytokines in pituitary adenomas.

However, a case report of an aggressive hormone‐secreting pituitary adenoma showed high levels of CD8+ lymphocyte infiltration in tumor tissue. The author suggested that the increase in tumor invasiveness and drug resistance in this case may be related to the infiltration of CD8+ lymphocytes^53^ (Figure 2A).

In addition, refractory pituitary adenomas also have something in common with nonrefractory pituitary adenomas. In one study, the Th1/Th2 ratio was significantly reduced after surgery, indicating cross‐regulation among Th cell subsets, and playing an important role in the inflammatory lesions of pituitary adenoma tissue^54^ (Figure 2B).

In conclusion, the presence and proportion of regulatory T lymphocytes and cytotoxic T lymphocytes in the pituitary adenoma microenvironment are largely related to the invasion ability, drug resistance, and other biological characteristics of pituitary adenoma. Therefore, it is very important to conduct in‐depth studies on each subgroup of T lymphocytes in the refractory pituitary adenoma microenvironment. It may profoundly affect the future immune diagnosis and treatment of refractory pituitary adenoma.

### 4.3 Tumor‐associated fibroblast

TAFs are the most abundant population in the tumor stroma of refractory pituitary adenomas. Invasive pituitary adenoma‐derived TAFs have been shown to significantly promote the growth of rat pituitary cells both in vitro and in vivo, and VEGF was overexpressed in both invasive pituitary adenoma‐derived TAFs and tumor specimens.^55^ In addition, the proliferation, migration, and invasion of human pituitary adenoma cells were inhibited by small‐interfering RNA‐mediating fibroblast growth factor gene silencing, further confirming the role of TAF and fibroblast growth factor in various biological behaviors of refractory pituitary tumors.^56^ Although there are few studies on TAF in pituitary adenoma at present, it can be found that TAF is an important part of the pituitary adenoma microenvironment through the above research reports. Therefore, future studies are still needed to further analyze the relationship between TAF and malignant phenotype of refractory pituitary adenoma.

## 5 NONCELLULAR COMPONENTS OF THE IMMUNE MICROENVIRONMENT

### 5.1 Extracellular matrix

The ECM plays an important role in tumor invasion and metastasis and may be related to the formation of refractory pituitary adenomas.^57^ The function of the ECM is affected by the dynamic balance between MMPs (matrix metalloproteinase) and TIMPs (tissue inhibitor of matrix metalloproteinases), and a systematic review and meta‐analysis involving 1320 patients with pituitary adenomas found elevated expression levels of MMP‐9 and MMP‐2 in patients with invasive pituitary adenomas, but no difference in TIMPs.^57^ Further studies are needed to confirm these results.

### 5.2 PD‐1/PD‐L1 axis

PD‐1/PD‐L1 axis plays a key role in inhibiting antitumor immunity. PD‐L1 transcription levels were significantly higher in primary pituitary adenomas than in recurrent pituitary adenomas.^58^ In addition, PD‐1/PD‐L1 was also associated with the invasiveness of pituitary adenomas. In one study, the expression of PD‐L1 was higher in invasive pituitary adenomas than in pituitary adenomas without invasive behavior.^59^ Therefore, more studies are needed on the relationship and mechanism between PD‐1/PD‐L1 axis and immune escape of pituitary adenoma.

### 5.3 Cytokines

**FIGURE 3:**
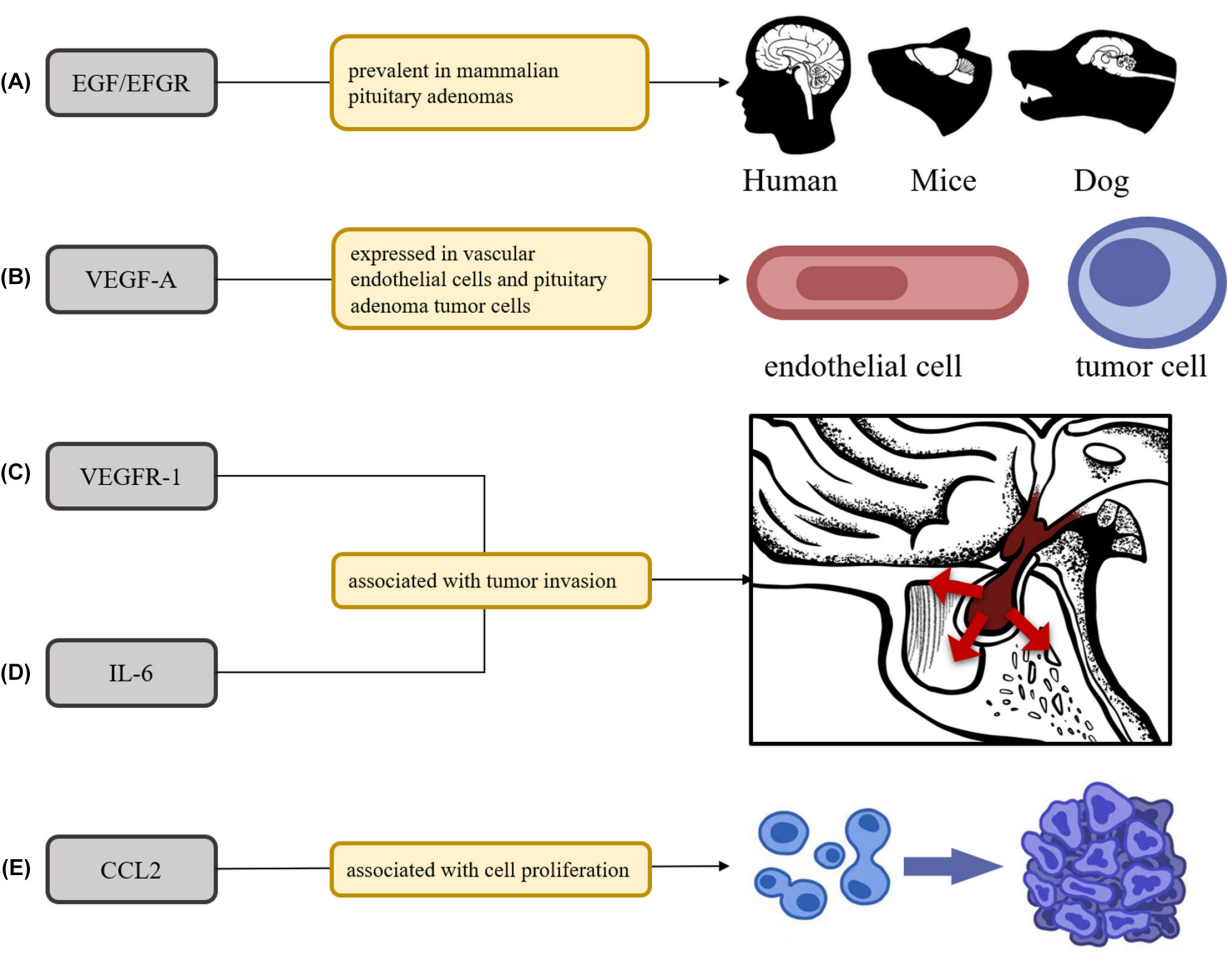
Effect of cytokines on immune microenvironment of pituitary adenoma. (A) Epidermal growth factor (EGF) and its receptor (EGFR) are prevalent in mammalian pituitary adenomas, including in humans, mice, and dogs. (B) VEGF‐A is present in vascular endothelial cells and pituitary adenoma tumor cells. (C, D) VEGFR‐1 and IL‐6 may be related to the invasive behavior of pituitary adenoma. (E) CCL‐2 may be related to the proliferative behavior of pituitary adenoma.

Cytokines play a key role in various physiological and pathological activities of cells. Epidermal growth factor (EGF) and its receptor (EGFR) are prevalent in mammalian pituitary adenomas, including in humans, mice, and dogs^60^ (Figure 3A), and are expressed in different ways in all categories of human pituitary adenomas.^61^, ^62^

Vascular endothelial growth factor (VEGF) plays a key role in immunosuppressive microenvironments by inhibiting dendritic cell maturation.^63^ Studies have confirmed that VEGF‐A is not only expressed in vascular endothelial cells, but also in tumor cells in pituitary adenomas (Figure 3B), while VEGF‐A/VEGF receptor (VEGFR) 1 expression was significantly upregulated in nonfunctional pituitary adenomas invading the cavernous sinus (Figure 3C), suggesting a positive role of VEGF and its receptors in the development and invasion of pituitary adenomas.^59^, ^64^ In addition, the TAF‐related cytokines IL‐6 and CCL2 mentioned above were also positively correlated with the aggressiveness and proliferation index of pituitary adenoma, respectively^65^ (Figure 3D,E).

In summary, IL‐6, CCL2, EGF/EGFR, VEGF/VEGFR, and other cytokines are expressed in pituitary adenomas and are generally related to their aggressiveness, occurrence, and development. Immunotherapy targeting these cytokines may thus represent an important future direction of immune checkpoint therapy for pituitary adenomas.

### 5.4 Cytoskeleton

Cytoskeleton is a complex multifunctional network of dynamic protein filaments. It plays an important role in maintaining the cell shape and structure, and in cell movement, differentiation, and intracellular transport.^66^ A recent study confirmed that co‐expression of intermediate filament glial fibrillary acidic protein and cytokeratin in pituitary adenomas and pituitary cells was associated with hormone expression and low recurrence rates.^67^ The recurrence rate is also one of the important differences between refractory pituitary adenomas and nonrefractory pituitary adenomas.

### 5.5 Neuropeptides

Downregulation of GAL and GAL receptors was found in some pituitary adenoma tumor tissue samples, and expression levels in different subtypes of pituitary adenomas were negatively correlated with tumor proliferation.^68^ GAL‐positive subtypes tended to have higher cure rates, in accordance with similar results observed for GAL and its receptors in gliomas.^68^

### 5.6 Hormones and their receptors

Abnormal hormone secretion is one of the important signs of refractory pituitary adenoma. A study found that expression levels of FSH receptors were significantly higher in invasive pituitary adenomas (68%) compared with noninvasive pituitary adenomas (12%), suggesting that FSH receptors may be a marker of aggressiveness in pituitary adenomas.^69^

The differences and similarities between refractory pituitary adenoma and pituitary adenoma tumor immune microenvironment at the cellular and molecular levels are summarized in Table 2.

## 6 BIOLOGICAL BEHAVIORS RELATED TO THE IMMUNE MICROENVIRONMENT

The immune microenvironment is closely related to the size, infiltration ability, hormone‐secretion ability, and other specific biological behaviors of pituitary adenomas^12^, ^70^ (Table 3).

**TABLE 3:** Distinction between refractory pituitary adenoma and pituitary adenoma (2).

	Refractory pituitary adenoma	Pituitary adenoma
Response to conventional treatment	Resistant	Curable in most cases
Postoperative status	Relapse or regeneration	Curable in most cases
Tumor growth rate	Faster	Slower
Epidemiology	About 0.5% of pituitary adenomas	10%–25% of all intracranial tumors
Biological behavior		
Bone invasion	Bone destruction and invasion are typical biological behaviors of invasive (refractory) pituitary adenoma	–
Angiogenesis	EGFL7 is widely involved in angiogenesis, tumor growth, invasion, and metastasis of (GHPA)	Angiogenic mimicry occurs in pituitary adenomas
Recurrence and invasion	Recurrence and invasion are typical clinical manifestations of invasive (refractory) pituitary adenoma	Tends not to happen

### 6.1 Bone invasion

Bone invasion and destruction are typical biological behaviors of invasive pituitary adenomas, with bone‐invasive pituitary adenomas accounting for 6.4% of all pituitary adenomas.^71^

Cathepsin K plays an important role in bone resorption and has been shown to be expressed in some malignant tumors.^72^, ^73^, ^74^, ^75^, ^76^ Some authors thus believe that cathepsin K may also be indispensable for the bone‐destruction behavior of pituitary adenomas.^77^ However, further studies are needed to clarify this issue.

Regarding the mechanism of bone damage, Zhu et al. reported that MEG8 promotes TNF‐α expression by sponging miR‐454‐3p, which ultimately leads to the occurrence of bone destruction, and was associated with poor progression‐free survival.^78^ Another study explored the mechanism of bone invasion in pituitary adenoma tumor cells in vitro and found that GT1.1 cells activated the phosphoinositide 3‐kinase‐Akt, mitogen‐activated protein kinase, and calcium signaling pathways through the *RASSF10* (Ras Association Domain Family Member 10)‐*MDM2* (*MDM2* Proto‐Oncogene) pathway, and promoted the differentiation of RAW264.7 cells into osteoclasts through exosomes, resulting in bone destruction.^79^ Zhang et al. found that bone destruction required the involvement of osteopontin secreted by fibroblasts, which may lead to bone destruction in patients with NFPA by altering the expression and polymerization of caldesmon in fibroblasts.^80^

### 6.2 Angiogenesis

The continuous division and proliferation of tumor cells require a large amount of oxygen and nutrients, which rapidly leads to the impoverishment and anoxia of the microenvironment. In order to survive under unfavorable conditions, tumor cells thus need to adapt and adjust to the environment, to develop into a more malignant state. During this process, blood vessel‐like formation occurs within the tumor tissue.^81^ Numerous studies have investigated the hypoxic environment and key factors such as angiogenesis in gliomas.^82^ Human pituitary adenomas are generally considered to demonstrate more active angiogenesis than normal healthy pituitary tissue,^83^ and angiogenesis in pituitary tumors is mainly regulated by PGF (Placental Growth Factor) and VEGF.^84^ Studies have demonstrated that epidermal growth factor‐like domain 7 (EGFL7) is widely involved in GHPAs (hormone‐producing pituitary adenomas) angiogenesis, tumor growth, invasion, and metastasis and inhibits GHPAs growth and invasion by decreasing the expression of this molecule.^62^, ^85^

In addition, vasculogenic mimicry occurs in pituitary adenomas, which refers to the phenomenon in which vascular‐like channels, which are not lined by endothelial cells, are formed in tumors. Vasculogenic mimicry is seen in both recurrent and nonrecurrent nonfunctional pituitary adenomas, but not in normal pituitary tissues.^86^

### 6.3 Recurrence and invasion

Silent type III pituitary adenomas are more dangerous than conventional acellular adenomas in terms of the risks of invasion and recurrence.^51^ Whole‐transcriptome analysis showed that secreted proteins that inhibited T lymphocyte activity were upregulated in SS‐3 adenomas, while proteins associated with cytotoxic T lymphocyte antigen presentation were downregulated and the overall concentration of T lymphocytes was relatively reduced.^51^

Studies have confirmed that EFGR was overexpressed in recurrent growth hormone‐secreting pituitary adenomas and invasive silent subtype III adenomas, suggesting that EGF/EGFR expression may be related to the recurrence and aggressiveness of pituitary adenomas.^61^ Similar results were obtained in another study, which showed higher expression levels of EGFL7 and P‐EGFR in invasive growth hormone‐secreting pituitary adenomas than in noninvasive GHPA (growth hormone‐producing pituitary adenomas).^62^ RSUME (RWD‐containing sumoylation enhancer) plays an important role in pituitary adenoma invasion by stabilizing hypoxic‐inducing factor‐1α and promoting VEGFA expression.^87^ By comparing the expression levels of the above three factors in pituitary tissues, He et al. found that the expression levels of RSUME and hypoxic‐inducing factor‐1α receptor were significantly higher in invasive pituitary adenomas compared with noninvasive pituitary adenomas.^87^

The similarities and differences in the biological behavior of pituitary adenomas and refractory pituitary adenomas, and the relationship between these phenomena and the TME are summarized in Table 3.

## 7 CURRENT STATUS OF IMMUNOTHERAPY FOR PITUITARY ADENOMA

Surgical treatment is currently the first choice of treatment for pituitary adenomas and refractory pituitary adenomas; however, the postoperative recurrence rate of some tumors remains high.^18^, ^88^, ^89^ In addition to surgery and radiation therapy, temozolomide is currently the only oral chemotherapeutic drug considered to be effective for pituitary adenomas, and some studies have suggested that early treatment with temozolomide may also have positive effects in patients with functional invasive pituitary adenomas.^17^, ^90^

Immunotherapy is a promising treatment that has shown clinical activity in many cancer types.^91^, ^92^ Previous studies have explored pituitary adenomas and invasive pituitary adenomas at the molecular level, and have summarized the therapeutic targets and clinical experience.^93^ Many studies^94^, ^95^, ^96^ have shown that exosomes regulated the TME and were closely related to tumor immunotherapy, and corresponding studies and clinical trials have been conducted in patients with gliomas.^97^ These results thus provided a promising direction for further exploration of immunotherapy for pituitary adenomas. Lin et al. treated a female patient with an invasive ACTH‐pituitary adenoma with the combination of ipilimumab and nivolumab with good therapeutic effect and suggested that this combination might provide an effective treatment for pituitary cancer.^98^

In addition, DCs (dendritic cells) in invasive pituitary adenomas were shown to be activated by mucin 1 and polyinosinic:polycytidylic acid (poly I:C).^99^ Mucin 1 is an epithelial membrane‐associated glycoprotein and an important molecular marker, with obvious positive expression in invasive pituitary adenoma, while poly I:C is often used as an immune stimulant or adjuvant. In this study, mucin 1 + poly I:C stimulated the expression of co‐stimulatory molecules to polarize the immune response toward Th1, and ultimately triggered the progression of antitumor immunosuppressive pituitary tumors.^99^

The above summary indicated a lack of clinical applications relating to current immunotherapy research on pituitary adenomas, and there is currently no systematic treatment direction or strategy. Notably, however, attitudes toward immunotherapy remain positive, and further studies are needed to identify treatments that take account of the specific immune status of pituitary adenomas and refractory pituitary adenomas.

## 8 CONCLUSIONS

In conclusion, the immune microenvironments of pituitary adenomas and refractory pituitary adenomas are dynamic and complex, and influence the whole process of diagnosis, treatment, and prognosis of pituitary adenomas. Although the immune microenvironment and immunotherapy represent a promising direction for future exploration and research in relation to pituitary adenomas, relevant research is currently lacking and further studies are needed to explore this issue.

## AUTHOR CONTRIBUTIONS

Conception and design of the review: W.C, A.W., and Q.C. Drafting the manuscript and the figure: Y.L., X.R., W.G., and R.C. Modifying the manuscript critically for important content: J.W., T.L., X.C., D.J., and C.C. All authors contributed to the article and approved the submitted version.

## FUNDING INFORMATION

This work was supported by the National Natural Science Foundation of China (nos. 81902546, 82373342 to W. Cheng; U20A20380, 81172409, 81472360, and 81872054 to A. Wu); Liaoning Science and Technology Plan Projects (no. 2011225034 to A. Wu); National Postdoctoral Program for Innovative Talents (no. BX20180384 to W. Cheng); China Postdoctoral Science Foundation (no. 2019M651169 to W. Cheng); Liao Ning Revitalization Talents Program (no. XLYC1807255 to W. Cheng); Shenyang Municipal Science and Technology Bureau Medical‐Industrial Joint Project (no. 213958 to W. Cheng); Shenyang Young and Middle‐Aged Science and Technology Innovation Talents Project (no. RC220087 to W. Cheng); Liaoning Province unveiled the leading science and technology plan (major) projects (no. 2022JH1/10400004 to W. Cheng); Shengjing Hospital Affiliated to China Medical University 345 Talent Project 30A Level (2022) (no. 1000801592 to W. Cheng).

## CONFLICT OF INTEREST STATEMENT

Not applicable.

## CONSENT FOR PUBLICATION

Not applicable.

## Data Availability

Data sharing is not applicable to this article as no new data were created or analyzed in this study.
